# Synthetic Promoter Library for Modulation of Actinorhodin Production in *Streptomyces coelicolor* A3(2)

**DOI:** 10.1371/journal.pone.0099701

**Published:** 2014-06-25

**Authors:** Sujata Vijay Sohoni, Alessandro Fazio, Christopher T. Workman, Ivan Mijakovic, Anna Eliasson Lantz

**Affiliations:** 1 Department of Systems Biology, Denmark Technical University, Kongens Lyngby, Denmark; 2 Systems and Synthetic Biology, Department of Chemical and Biological Engineering, Chalmers University of Technology, Gothenburg, Sweden; Imperial College London, United Kingdom

## Abstract

The objective of this study was the application of the synthetic promoter library (SPL) technology for modulation of actinorhodin production in *Streptomyces coelicolor* A3(2). The SPL technology was used to optimize the expression of a pathway specific positive transcriptional regulator ActII orf4, which activates the transcription of the *S. coelicolor* actinorhodin biosynthetic gene cluster. The native *actII orf4* promoter was replaced with synthetic promoters, generating a *S. coelicolor* library with a broad range of expression levels of *actII orf4*. The resulting library was screened based on the yield of actinorhodin. Selected strains were further physiologically characterized. One of the strains from the library, ScoSPL20, showed considerably higher yield of actinorhodin and final actinorhodin titer, compared to *S. coelicolor* wild type and *S. coelicolor* with *actII orf4* expressed from a strong constitutive promoter. ScoSPL20 demonstrated exceptional productivity despite having a comparatively weak expression from the promoter. Interestingly, the ScoSPL20 promoter was activated at a much earlier stage of growth compared to the wild type, demonstrating the advantage of fine-tuning and temporal tuning of gene expression in metabolic engineering. Transcriptome studies were performed in exponential and actinorhodin-producing phase of growth to compare gene expression between ScoSPL20 and the wild type. To our knowledge, this is the first successful application of the SPL technology for secondary metabolite production in filamentous bacteria.

## Introduction

Streptomycetes produce more than two thirds of all commercially available antibiotics. They are soil-dwelling bacteria, included in the taxonomic order *Actinomycetales* and have a complex life cycle switching between the vegetative hyphae and the spores. *Streptomyces coelicolor* A3(2) (WT) is the best characterized member of the genus *Streptomyces*.

*S. coelicolor* produces four biochemically well characterized antibiotics, namely a blue colored polyketide actinorhodin (ACT) [1], undecylprodigiosin also known as RED [2], methylenomycin [3] and a Ca-dependent antibiotic [4]. Production of these antibiotics is regulated at different levels; the ACT biosynthesis being the most studied one. ACT production has been shown to be influenced by a number of genes involved in the morphological differentiation (*bld* genes), as well as genes that have pleiotropic effects (*afs, rel, abs* and *dasR* family of genes). Deletion or overexpression of one of the fore-mentioned pleiotropic-effect genes has been demonstrated to result in overproduction of ACT [5], [6], [7], [8]. Furthermore, the transcriptional regulator AtrA was shown to directly bind to the promoter region of *actII orf4* and initiate ACT production [9]. Cyclic AMP receptor protein (Crp) has been identified as a global regulator of secondary metabolism by controlling production of precursors. The expression of six secondary metabolic pathways in S. coelicolor was affected by Crp and overexpression of Crp also led to increased antibiotic production in other Streptomyces [10]. In addition, a pathway specific regulator encoded by *actII orf4* has been identified as a positive regulator of the ACT gene cluster [11], [12].

Various strategies of gene manipulation have been applied on improving properties of industrial microorganisms. Most of these approaches involve overexpression of genes encoding rate-limiting enzymes or inactivation of competing branches of branched pathways [13], [14]. Such strategies have been fruitful in some cases [15], [16], [17], but can fall short when discrete optimization of gene expression is needed to fine-tune the modified pathway [18]. Promoter strength plays an important role in the resulting levels of gene expression [14], [19]. The synthetic promoter technology, based on randomization of promoter sequences, has been successfully employed to construct promoter libraries for optimized levels of gene expression [14], [19], [20]. To list a few examples, the synthetic promoter library (SPL) approach has been used for fine-tuning of expression of recombinant proteins in *E. coli* [21], for metabolic engineering in *Lactococcus lactis* [22], for constitutive gene expression in *Lactobacillus plantarum* [23] and for improving lycopene production in *S. cerevisiae* [19]. Along similar lines, Seghezzi et al. (2011) reported the construction of a generalized synthetic promoter library in *Streptomyces lividans* [24].

The aim of our study was to investigate how modulation of expression levels of the positive regulator *actII orf4* affects ACT production in *S. coelicolor*. In a benchmark strain, the expression of *actII orf4* was enhanced by constitutive overexpression (oxp) under control of the promoter ermE*. In parallel, a SPL was constructed based on the randomization of the sequences flanking the native promoter of *actII orf4*, which enabled us to select a *S. coelicolor* A3(2) strain with even higher yield and productivity of ACT than the benchmark strain, with constitutively overexpressed *actII orf4*. Moreover, a transcriptome analysis was undertaken for the high-yielding strain to assess the global extent of deregulation caused by the replacement of the native *actII orf4* promoter. To the best of our knowledge, this is the first successful application of the SPL technology in filamentous bacteria for modulation of antibiotic production. Some of the promoters obtained in this study underwent further characterization to make them a generally available tool for tuning of gene expression in *Streptomyces* spp.

## Materials and Methods

All the chemicals used in this study were analytical grade unless otherwise stated. All solvents were HPLC grade. All the chemicals and solvents were purchased from Sigma–Aldrich (Steinheim, Germany). Water (MQ) was purified with a Milli-Q-system (Millipore, Bradford, MA). Microtiter plates and lids were obtained from Enzyscreen (Leiden, Netherlands). Bacterial strains and plasmids are documented in Table S1 in File S1.

### Media and growth conditions for strain construction

*E. coli* cultures were grown at 37°C in Luria Bertani broth [25]. Gentamycin (25 µg ml^−1^) and ampicillin (100 µg ml^−1^) were used for *E. coli* DH5α with containing the plasmid pGM160. Kanamycin (25 µg ml^−1^) and choloramphenicol (25 µg ml^−1^) were used for *E. coli* with ET12567/pUZ8002. Nalidixic acid (20 µg ml^−1^) was used for *E. coli*. Hygromycin (50 µg ml^−1^) was used for *E. coli* with the plasmid pIJ10257 and the *actII orf4* overexpression strain of *S. coelicolor*. Thiostrepton (25 µg ml^−1^) was used for *S. coelicolor*.

Mannitol-Soya flour (MS) agar was used for plating *S. coelicolor*. 2X YT (16 g l^−1^ tryptone, 10 g l^−1^ yeast extract and 10 g l^−1^ sodium chloride) medium was used for germination of spores.

### DNA related techniques

DNA techniques involving *E. coli* were performed as described by Sambrook *et al*. (2001) [25]. *E. coli* transformations were performed using electroporation. Expand high fidelity Taq polymerase purchased from Roche diagnostics (Germany) was applied for PCR intended for cloning. Taq polymerase purchased from Sigma Aldrich (USA) was applied for analytical purposes. Chromosomal DNA from *S. coelicolor* was isolated as described by Kisser *et al.* (2000) [26]. *S. coelicolor* spores were streaked on MS agar plates to obtain fresh spores. The spores were harvested in 20% glycerol and used for conjugation.

### Construction of the *actII orf4* overexpression strain (*S. coelicolor* oxp-*actII orf4*)

The gene *actII orf4* was amplified using a forward primer with a *NdeI* site and a reverse primer with a *PacI* site (primer sequences described in Table S2 in File S1) and inserted into the plasmid pIJ10257. The resulting plasmid pIJ10257_*actII orf4* was used to transform *E. coli* ET12567/pUZ8002. *E. coli* containing ET12567 pIJ10257_*actII orf4* was then conjugated with *S. coelicolor* WT, and the hygromycin-resistant colonies were selected.

### Construction of the promoter library

First the upstream region of *actII orf 4* gene was amplified from *S. coelicolor* genomic DNA using a forward primer, 5′-TAGTGGATCCGGTGTCCGGCCGTCCGCGTGCCGTCTG -3′ with a *BamHI* restriction site (underlined) and a reverse primer 5′ – TTCGTCTTAATTAACTACTGCACATATGTACTTCTAGAGCCGTCACAAGCGATCTCCTATTG -3′ with a *XbaI* site (underlined). Restriction sites for *NdeI* and *PacI* (underlined) were also included in the reverse primer. The *NdeI* and *PacI* were included to facilitate the insertion of the *actII orf4* gene in the following step. The PCR product was digested with *BamHI* and *XbaI* and inserted in the plasmid pGM160. This construct was named pGM160_ups_*actII orf4*. A second PCR fragment was generated for the *actII orf4* gene using a forward primer, 5′- GTCATATGCTTGANNNNNTTGAAANNNNNNNNNNNNNNNNNTTATTNNNNNAGCTGTACAGGGGACAGCTGGGACACCCAAGGAAGAAGGCTGACGTCCGACATGAGATTCAACTTATTGGGACGTG-3′ bearing a *NdeI* site, and a reverse primer 5′- ATGATTAATTAACTACACGAGCACCTTCTCAC-3′ with a *PacI* site. The resulting PCR product was digested with *NdeI* and *PacI*, and inserted between the same restriction sites of the previously constructed plasmid pGM160_ups_*actII orf4*, containing a 1.126 kb upstream region of *actII orf4* gene, to obtain the plasmid pGM160_SPL. *ActII orf4* is naturally only transcribed in the stationary phase and hence in order to ensure the stability of the transcript, the leader sequence of the native gene was replaced with that of a glycolytic gene *pgi2* [14], [27]. This was done by including the *pgi2* leader sequence in the forward primer (underlined in the primer) for amplification of *actII orf4*. Plasmid pGM160_SPL was used to replace the native promoter of *actII orf 4* with the synthetic ones, thus generating the SPL strains. A scheme representing various steps of the library construction is included in Figure S1.

The plasmid pGM160_SPL was then transferred into *S. coelicolor* through conjugation with *E. coli* ET12567/pUZ8002. After a 30 hours incubation at 30°C, the conjugation plates were transferred to 37°C in order to eliminate autonomously replicating plasmids [28]. The conjugation plates were incubated at 37°C until visible colonies of *S. coelicolor* appeared (around 3–4 days). The colonies with double cross-overs were selected by plating them for 4–5 generations. Details are mentioned in Figure S2.

The chosen SPL promoters were sequenced using the forward primer 5′-CGATGTCGGCCGGTGGATGTG-3′ and the reverse primer 5′-ACACGTACGTCTGCAGCGTCGTCATG-3′.

### Frozen mycelium stock preparation for SPL colonies

Frozen mycelium stock was prepared for 200 *Streptomyces* colonies chosen for screening (For details see Text S1 in File S1). Frozen mycelium stocks for *S. coelicolor* WT strain, *S. coelicolor* oxp-*actII orf4* and the 11 SPL strains chosen for detailed physiological characterization were prepared by inoculating spore stocks prepared as described above, in 50 ml of the 2X YT medium and grown in 500 ml shake flasks, following the procedure described by Sohoni *et al*. (2012) [29].

### Initial characterization of the SPL in minimal medium

The defined minimal medium used for all the cultivations and characterization of the SPL strains was adapted and modified from the phosphate limited Evan's medium (1970). The medium was prepared as described by Sohoni *et al*. (2012), details are described in Text S2 in File S1) [29]. *S. coelicolor* cultivations in microtiter plates and shake flasks were performed at 28°C, with 150 rpm agitation, as described by Sohoni *et al*. (2012) [29]. 20 µl of each frozen stock was inoculated in 10 ml of defined minimal medium. This was then distributed in 24 well microtiter plates with 3 ml working volume, with one well representing a single cultivation. All the colonies were inoculated in duplicates. OD_450nm_ was measured throughout the experiment. After 120 hours, cultivation was terminated and ACT production, biomass (dry cell weight/DCW), glucose content, and RED production were measured as described below.

### Detailed characterization of selected SPL strains in microtiter plates and bioreactors

11 strains were selected for detailed physiological characterization in microtiter plates. In addition, ScoSPL20 was characterized in bioreactors along with the reference strains *S. coelicolor* WT and the benchmark production strain oxp-*actII orf4*. Batch cultivations in microtiter plates using the minimal medium were performed in duplicates and as described by Sohoni *et al*. (2012) [29]. Samples from microtiter plates were taken at different time points and analyzed for OD_450nm_, DCW, RED production, ACT production, pH and glucose content. Batch cultivations in bioreactors were performed at 28°C, pH 6.8–6.9, 500 rpm agitation rate and a 1 vvm aeration rate. Applikon bioreactors with 1 l working volume were used in this study (Applikon Biotechnology, Schiedam, Netherlands). Minimal medium excluding MOPS buffer was inoculated with 1 ml of the frozen mycelia stock. 2 M NaOH was used to maintain the pH. An acoustic gas analyzer was used to monitor the concentrations of CO_2_ and O_2_ in the exhaust gas (1311, Innova Air Tech Instruments A/S. Nærum, Denmark). Samples taken from bioreactors at different time points were analyzed for DCW, RED production, ACT production and glucose content.

### Dry cell weight (DCW) analyses

2.2 ml of the fermentation broth were filtered and analyzed as described by Borodina *et al*. (2008) [17].

### ACT and RED measurements

For ACT production measurements, 100 µl of broth was mixed with 1.9 ml of 2 M NaOH. For RED production measurements, 100 µl of broth was mixed with 1.9 ml methanol-HCl (pH 1.5). Measurements were performed as described by Borodina *et al*. (2008) [17].

### Glucose measurements

HPLC method described by Borodina *et al*. (2008) was used for measuring the glucose content in the medium [17].

### Determination of promoter expression levels

The xyloglucanase gene (*xeg*) from *Jonesia* sp, optimized for extracellular secretion in *S.lividans* by Sianidis *et al.* (2006) [30], was used as a reporter gene to quantify the expression from the sequenced synthetic promoters. Synthetic promoters were incorporated in front of the *xeg* gene using a forward primer containing a *XbaI* site and a reverse primer with a *NcoI* site (primer sequences can be found in Table S3 in File S1). Respective promoter-xeg products were inserted into the plasmid pGM160 (can also function as an expression vector when temperature is not raised above 34°C). The resulting pGM160_promoter_xeg plasmids were used to transform *E. coli* ET12567/pUZ8002, which eventually was conjugated with *S. coelicolor*. Thiostrepton resistant colonies were selected.

Frozen mycelia stocks were prepared in microtiter plates for all the Sco-promoter-xeg colonies as described by Sohoni *et al*. (2012) [29]. These were subsequently grown in a modified Evan's medium (Text 4), using microtiter plates as described above. Samples from all the strains were collected at OD_450nm_ 6.0. Xeg activity was determined as described by Sianidis *et al*. (2006) [30].

### Transcriptome analysis

Transcriptome analysis was performed for the WT strain and the ScoSPL20 strain in the mid-exponential phase and in the ACT-producing phase, using custom made Affymetrix chips for *S. coelicolor* A3(2). Samples in the mid-exponential phase were collected after 21 h of growth for the ScoSPL20 strain and after 25 h of growth for the WT strain (for both the strains corresponding to a dry weight of approximately 0.5 g/L). Samples in the ACT-producing phase for the ScoSPL20 were collected after 52 h of growth and for the WT strain after 90 h. Detailed protocol for sampling, RNA isolation, probe preparation and hybridization is provided in Text S3 in File S1.

Affymetrix Microarray Suite v5.0 was used to generate the CEL files of the scanned DNA microarrays [31]. The statistical open source language R was used to perform data analysis [32]. Data preprocessing was carried out using the R/affy package [33], which implements the Robust Multichip Average (RMA) method by correcting the Perfect Match (PM) probes, performing quantile normalization [34] and calculating the expression measure by using median polish. Pair-wise comparisons using moderated *t*-test implemented in the R/Limma package were carried out between the conditions, in order to detect differential transcriptional regulation [35]. A *P*-value of 0.05 was chosen as cut-off for significance and multiple correction was performed according to the Benjamini and Hochberg methodology [36].

## Results

### Construction and screening of a SPL for modulation of ACT production

**Figure 1:**
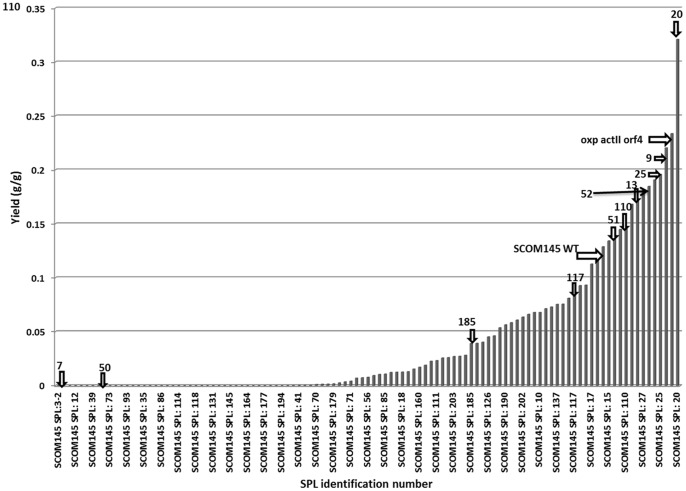
Initial screening of Synthetic Promoter Library. Yield of ACT (g/g) on biomass for 200 selected SPL strains at the end of 120 h of cultivation. *S. coelicolor* WT strain and *S. coelicolor* oxp-*actII orf4* were used as reference strains. Arrows and numbers on the yield graph indicate the position of 11 ScoSPL strains that were selected for detailed physiological characterization. Number of experimental replicates: 2.

A SPL was constructed for modulation of ACT synthesis in *S. coelicolor* using the native *actII orf4* promoter as a target, and randomizing the regions flanking the consensus sequences. Around 10,000 colonies were screened by visual inspection, and 200 colonies showing a dark blue halo of ACT were selected for further characterization. For each selected colony, duplicate cultivations were performed in microtiter plates and biomass, ACT production, RED production and glucose content were analyzed after an 120 h incubation. *S. coelicolor* WT was included in the characterization as reference. The final yield of ACT on biomass (g/g) was chosen as a criterion for comparison of different strains. A broad span of yields was obtained, spanning both above and below the WT level (Figure 1). However, some of the selected 200 strains failed to grow in the minimal medium. The strain denoted ScoSPL20 had the highest absolute ACT yield, approximately 2.8 times higher than that of the WT strain. ScoSPL20 and an additional 10 randomly selected SPL strains having higher or lower yields of ACT on biomass were chosen for a detailed physiological analysis.

### Further physiological characterization of eleven selected SPL strains

Of the selected strains, ScoSPL9, ScoSPL20 ScoSPL25, ScoSPL51, ScoSPL52, ScoSPL110 showed higher yields of ACT, while ScoSPL7, ScoSPL13, ScoSPL50, ScoSPL117 and ScoSPL185 showed lower yields of ACT compared to the WT strain. ScoSPL7, ScoSPL50 and ScoSPL185 did not produce any ACT up to 120 h of cultivation. The strains were analyzed in in microtiter plates. The measured parameters included OD_450nm_, dry cell weight (g l^−1^), glucose content (g l^−1^), ACT production (mg l^−1^) and RED production (mg l^−1^). Since this type of characterization routinely gives very reproducible results in our experimental setup (within 5% relative error); all measurements were performed in two, rather than three, biological replicates. The rates and yields calculated from one replicate for the characterized SPL strains and the *S. coelicolor* WT are presented in Table 1.

**Table 1:** Comparative table representing specific growth rate (μ), volumetric production rate for AC (q_P_ACT) and volumetric production rates for undecylprodigiosin (q_P_RED) represented as mg l^−1^ h^−1^ and yield of biomass over substrate (Y_SX_) from one of the two biological replicates.

ScoSPL ID	μ (h^−1^)	_qP_ACT (mg l^−1^ h^−1^)	_qP_RED (mg l^−1^ h^−1^)	Y_SX_ ♦ (g g^−1^)
**Sco (WT)**	0.11	9.4	1.82	0.32
**ScoSPL20**	0.14	35.96	1.82	0.27
**ScoSPL9**	0.12	16.7	0.23	0.26
**ScoSPL25**	0.15	9.32	0.38	0.36
**ScoSPL110**	0.13	8.89	0.09	0.13
**ScoSPL51**	0.14	7.68	0.08	0.22
**ScoSPL52**	0.13	7.19	0.1	0.2
**ScoSPL13**	0.15	7.16	0.47	0.24
**ScoSPL117**	0.12	6.9	0.1	0.26
**ScoSPL7**	0.14	0	0.31	0.21
**ScoSPL50**	0.11	0	0.06	0.26
**ScoSPL185**	0.12	0	0.06	0.26

♦ - Y_SX_ was calculated in the exponential phase of growth.

All the selected strains grew faster than the WT, except for the ScoSPL50 which grew at a rate comparable with that of the WT. Volumetric productivity of ACT (q_P_ACT) and yield of biomass on substrate (Y_SX_) for the ScoSPL strains were comparable to values obtained for the WT, except for ScoSPL9 that showed elevated q_P_ACT (almost double) and ScoSPL20 that showed elevated levels of both q_P_ACT and Y_SX_. There was a substantial decrease in the volumetric productivity of RED (q_P_RED) for all the ScoSPL strains, except for the ScoSPL20, for which the q_P_RED was comparable with that detected for the WT. Interestingly, all ScoSPL strains that displayed a higher yield of ACT on biomass (except the ScoSPL20), showed a distinct period in the RED phase where the biomass was constant, i.e. the growth was stalled for 12–15 h (data not shown). Among the 11 strains characterized, the ScoSPL20 was chosen for further analysis in bioreactors as the most promising one.

### Physiological characterization of reference strains in bioreactors

**Figure 2:**
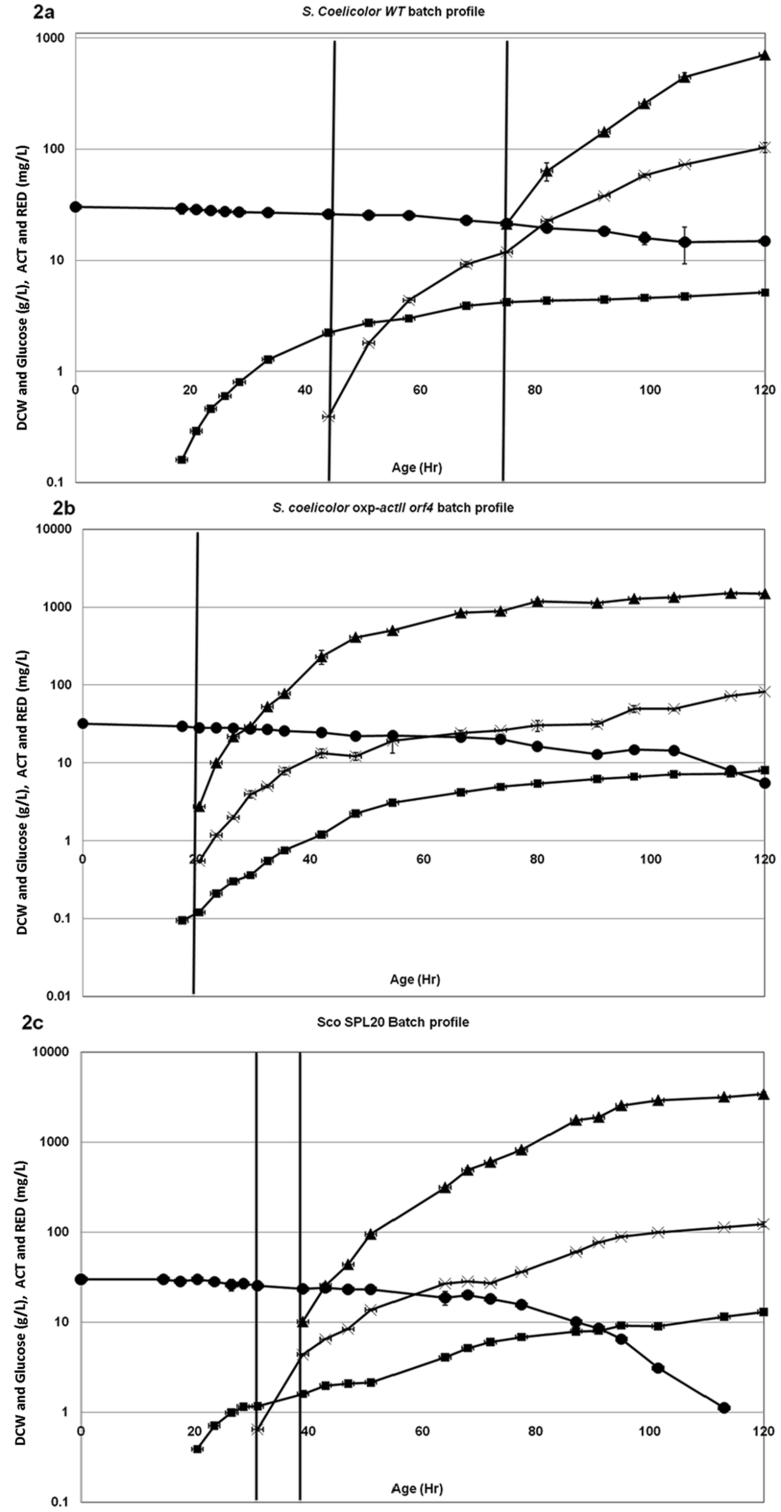
Batch profile of different strains in minimal medium, using a bioreactor. (A) Batch profile of *S. coelicolor* A3(2). The graph shows time course measurement for DCW (▪), glucose content in g l^−1^ (•), ACT production (▴) and RED production (×) in mg l^−1^. Vertical lines indicate the onset of RED and ACT production, respectively. Number of experimental replicates: 3. (B) Batch profile of *S. coelicolor* oxp-*actII orf4*. The graph shows time course measurement for DCW (▪), glucose content in g l^−1^ (•), ACT production (▴) and RED production (×) in mg l^−1^. Vertical line indicates the onset of RED and ACT production. Number of experimental replicates: 3. (C) Batch profile of ScoSPL20. The graph shows time course measurement for DCW (▪), glucose content in g l^−1^ (•), ACT production (▴) and RED production (×) in mg l^−1^. Vertical lines indicate the onset of RED and ACT production, respectively. Number of experimental replicates: 3.

*S. coelicolor* WT and *S. coelicolor oxp-actII orf4* were used as reference strains for comparative studies. Physiological characterization of the strains was performed in bioreactors in triplicate. Growth behavior of all the three strains is depicted in figure 2.

Typical growth behavior of the WT strain illustrates four different phases (Figure 2a), namely: 1) the lag phase (before sampling starts up to 20 h), 2) the exponential (exp) phase where the organism grows with maximum specific growth rate, 3) the RED-production phase or a second growth phase, where RED production starts and the culture continues to grow but at a reduced rate, 4) the stationary (stat) phase, where growth almost stops and production of ACT starts. ACT production continues until glucose in the medium is exhausted.

Cultivation of the oxp-*actII orf4* strain also displayed 4 phases, but with different properties (Figure 2b). The lag phase was followed by an exponential phase with maximum specific growth rate. Since *actII orf4* was expressed under a constitutive promoter, ACT production started already in the exponential phase. The specific growth rate in this phase (μ_exp_) for oxp-*actII orf4* strain (0.11 h^−1^) was comparable to that of the WT strain (0.10 h^−1^). Surprisingly, RED production started in parallel with ACT production in this strain, indicating crosstalk between the two pathways. We also observed another growth phase with reduced rate (0.031 h^−1^, 22.5% lower than μ for the second phase for the WT strain). In the stationary phase, the growth almost ceased and the culture continued to produce RED and ACT. For oxp-*actII orf4* strain, the specific substrate uptake rate (−r_S_) increased by 26.9%, while the yield, Y_SX_, decreased by 14.5% compared to the WT strain (Table 2). This effect may be due to the competition for glucose between the production of biomass and antibiotics. The volumetric production rate for RED decreased by 60% while, that of ACT increased by 27.5% in the oxp-*actII orf4* strain compared to the WT strain (Table 2).

**Table 2:** Comparative table describing physiological parameters of </emph>***S. coelicolor*** **WT,** ***S. coelicolor*** **oxp-** ***actII orf4*** **and ScoSPL20. Results are the mean values with standard deviations from three biological replicates.**

	Sco (WT)	Sco oxp *actII orf4* (ermE*)	Sco SPL20
**μ exp phase (h^−1^)**	0.1±0.005	0.11±0.001	0.12±0.01
**μ RED phase (h^−1^)**	0.04±0.01	†	0.03±0.01
**μ Act phase (h^−1^)**	0	0	0.03±0.002
**-rs** ‡ **(mmol glu g DCW^−1^ h^−1^)**	1.8±0.11	2.3±0.02	2.5±0.10
**q_P_ACT (mg l^−1^ h^−1^)**	12.4±0.34	15.8±0.8	47.7±4.30
**q_P_RED (mg l^−1^ h^−1^)**	1.9±0.18	0.8±0.10	1.5±0.10
**Y_SX_** ‡ **(g g^−1^)**	0.31±0.02	0.26±0.01	0.29±0.01

† Did not display a defined RED production phase, onset of RED and ACT production was simultaneous and growth associated.

‡ r_S_ and Y_SX_ were calculated in the exponential growth phase.

### Physiological characterization of ScoSPL20

ScoSPL20 also showed 4 distinct growth phases (Figure 2c). There was no significant change in μ_exp_ when compared with the WT and the oxp-*actII orf4* strains. A RED-production phase followed the exponential growth phase. The specific growth rate in the RED-production phase was 30% lower than observed for the WT strain (0.028 and 0.04 h^−1^, respectively) (Table 2). In addition, the RED-production phase in the ScoSPL20 was almost 12 h shorter than in the WT (Figure 2a and 2c). In the ACT-production phase, the ScoSPL20 continued to grow with a μ of 0.028 hr^1^, contrary to the WT strain that ceased growing. In the ScoSPL20 strain, -r_S_ increased by 37% and 10% compared to the WT and the oxp-*actII orf4* strains, respectively. The yield of biomass on glucose (Y_SX_) decreased by 7.5% compared to the WT strain. Another marked difference between the *S. coelicolor* WT and the ScoSPL20 was the onset of antibiotic production. In the ScoSPL20, there was an early shift to RED production and from RED to ACT production compared to *the* WT strain. However the antibiotic production did not come as early and in parallel with growth as observed for the oxp-*actII orf4* strain (Figure 2a, 2b and 2c). Volumetric production rate for RED decreased by 29% compared with the WT strain, while there was a significant increase in ACT volumetric production rate in the ScoSPL20. Volumetric production rate of ACT increased by 285% and 257% compared to the WT and the oxp-*actII orf4* strains, respectively (Table 2).

### Transcriptome analysis of the ScoSPL20

**Figure 3:**
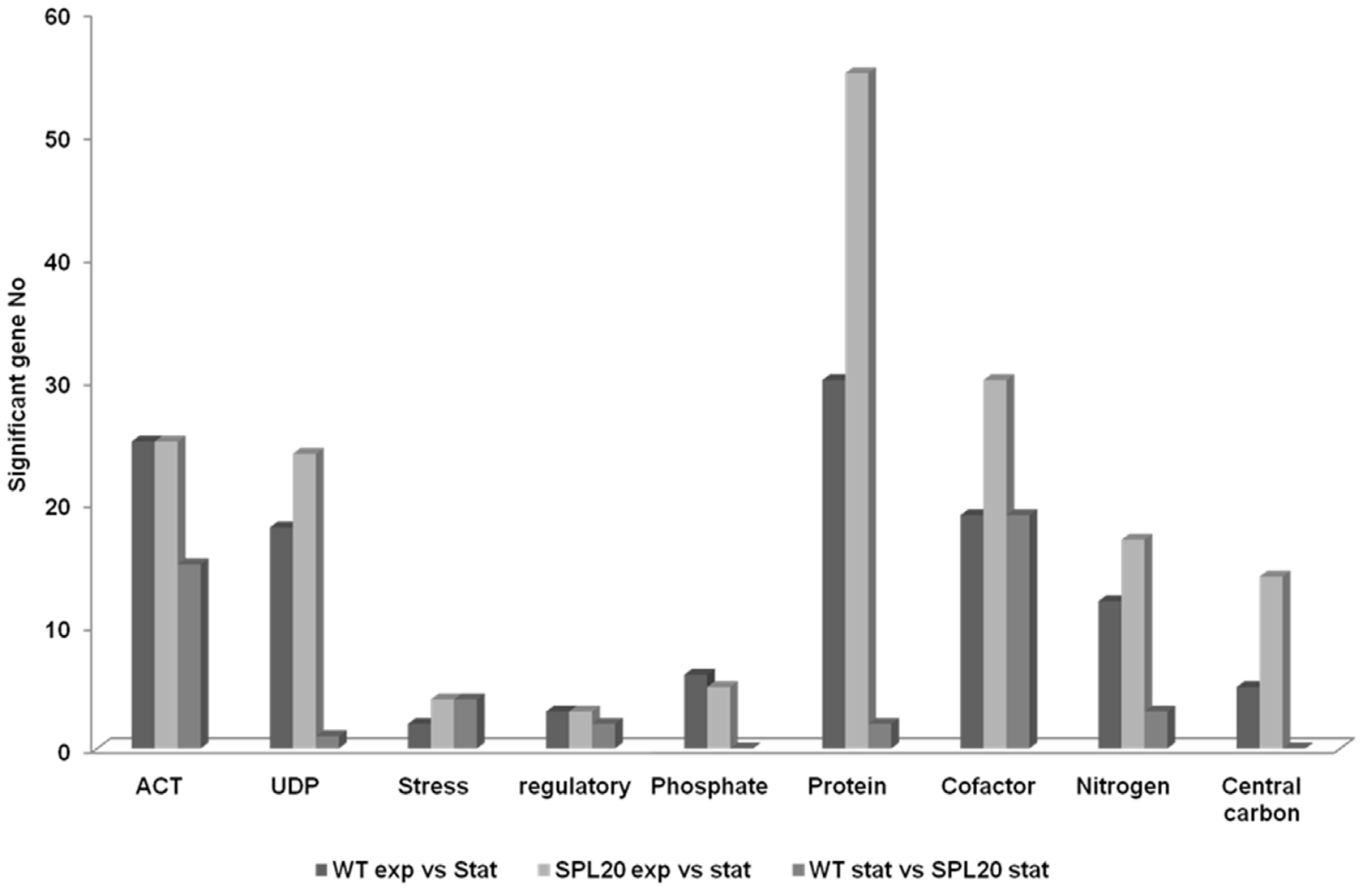
Summary of transcriptome analysis. Categories of genes that are up-regulated or down-regulated in the comparison of gene expression profiles of the WT and the ScoSPL20 strains in the exponential and stationary phases.

Transcriptome analysis was performed in mid-exponential phase and ACT production phase to compare gene expression profiles of the ScoSPL20 and the WT strain. Since the two strains differ in time course of the different growth and production phases, samples in the ACT phase were not collected at a specific time point but at a fixed ACT concentration of 100 mg/L i.e. when the cells were committed to ACT production. Gene expression profiles were compared intra-strain i.e. exponentially growing WT to ACT-producing WT and exponentially growing ScoSPL20 to ACT-producing ScoSPP20; and inter-strain i.e. exponentially growing WT to exponentially growing ScoSPL20 and ACT-producing WT to ACT-producing ScoSPL20, at P-value of 0.05. The genes that were significantly up-regulated or down-regulated were divided into 10 different groups, of which 9 are represented in Figure 3. The 10^th^ group, consisting of hypothetical and putative genes was omitted.

Comparison of exponentially growing WT to ACT-producing WT transcription profiles resulted in the identification of 299 genes with significant changes in expression, of which 194 were up-regulated and 105 were down-regulated in the stationary phase. The genes that were up-regulated included genes for biosynthesis of ACT (25 genes) and RED (18 genes). Furthermore, 6 genes involved in phosphate metabolism including *phoP* and *phoR* were up-regulated. Genes for glutamate and glutamine synthesis were down-regulated, while those for nitrate metabolism were up-regulated. With respect to cofactor metabolism, NADH dehydrogenase subunits were up-regulated, while cytochromes and ATP synthase were down-regulated. Thirty genes involved in ribosomal synthesis and 5 genes involved in central carbon metabolism were significantly down-regulated in the stationary phase.

Gene expression profile comparison of exponentially growing ScoSPL20 and ACT-producing ScoSPL20 resulted in detection of 449 genes with significant changes in expression. Of these, 185 genes were up-regulated and 264 genes were down-regulated. 25 genes for ACT biosynthesis and 24 genes for RED biosynthesis were up-regulated in the stationary phase. In addition, *afsR2* and 5 genes for phosphate metabolism were found to be up-regulated. 55 genes connected to ribosomal synthesis and protein synthesis, and 14 genes in central carbon metabolism were significantly down-regulated. Of the 30 genes significant for cofactor metabolism, NADH dehydrogenase subunit genes were up-regulated, while cytochrome and ATP synthase were down-regulated. As for the WT strain, genes for amino acid metabolism were down-regulated, while nitrate metabolism genes were up-regulated in the stationary phase.

Among the hypothetical and putative genes in the comparison of the exponentially growing WT to ACT-producing WT and the exponentially growing ScoSPL20 to ACT-producing ScoSPL20, some genes that were up-regulated are associated with phosphate metabolism.

**Figure 4:**
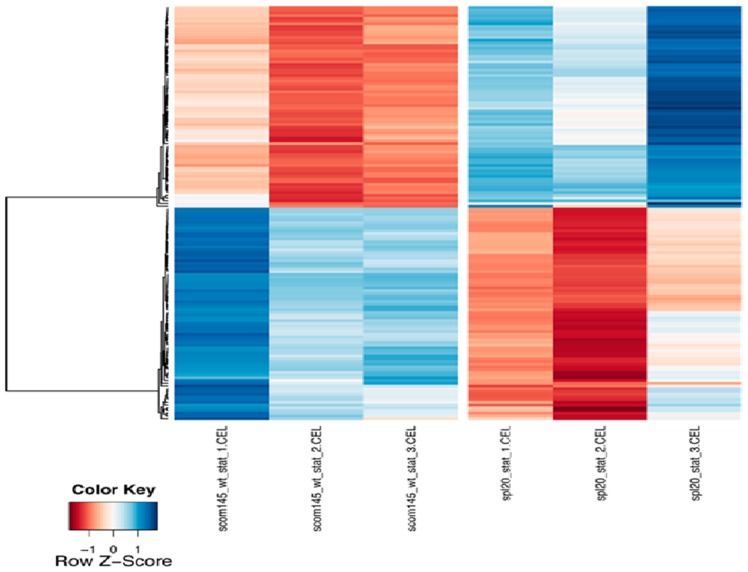
Hierarchical clustering (Pearson correlation, average distance) of the genes. Genes with a significant change in expression level between the WT and the SPL20 strain during the stationary phase. The rows and columns represent genes and samples respectively. The scale of the color-bar is based on the z-score.

Inter-strain comparison of exponentially growing WT and exponentially growing ScoSPL20 did not yield any genes with significant expression changes. However, the expression profile comparison of ACT-producing WT and ACT-producing ScoSPL20 resulted in 154 genes with significantly different expression, of which 75 were up-regulated and 79 were down-regulated (Figure 4) in the ScoSPL20. Of these genes, 15 ACT biosynthetic genes and 1 RED biosynthetic gene were up-regulated. 3 genes involved in glutamate metabolism, 3 genes involved in peroxide breakdown and 14 genes coding for the NADH dehydrogenase were also up-regulated in the ScoSPL20.

### Promoter sequencing and determination of expression levels

**Figure 5:**
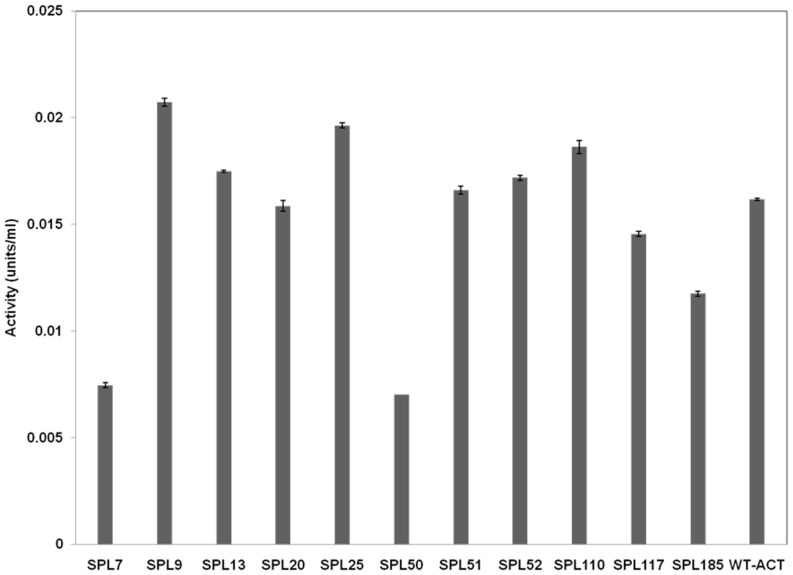
Expression levels of different SPL promoters – determined using *xeg* as reporter gene. WT-ACT refers to the native *actII orf4* promoter. Number of experimental replicates: 2.

Promoters for the 11 SPL strains that were studied in detail were sequenced. Table 3 represents sequences of the promoters of these SPL strains, with the wild type promoter sequence included for reference. Expression level from these promoters was measured using the xyloglucanase gene from *Jonesia* sp. (*xeg*) as a reporter. The expression level of different promoters is represented in Figure 5. A clear correlation between the Xeg activity and the yield of ACT on biomass was detected for most of the promoters (Figure 5; Table 2). The native *actII orf4* promoter activity was in between the highest and the lowest values of activities. Surprisingly, the expression level of the ScoSPL20 promoter, which gave the highest yield of ACT on biomass, was similar to that of the native *actII orf4* promoter. Furthermore the SPL20 promoter also exhibited some degree of variations with respect to the non-randomized promoter regions. This sort of variation in predefined PCR primer sequences often arises during SPL construction due to errors in the synthesis of such long oligonucleotides.

**Table 3:** Promoter sequences for selected ScoSPL strains and that of the wild type *actII orf4* promoter.

Strain Name	Promoter sequence
Synthetic promoter	NNNNN**TTGAAA**NNNNNNNNNNNNNNNNN**TTATT**NNNNN
*actII orf4* WT	GCACA**TTGAAA**TCTGTTGAGTAGGCCTG**TTATT**GTCGC
ScoSPL7	CGGGC**TGGGGC**TCGGGGCCGCCGGTGAG**CTGGT**AGACG
ScoSPL9	GCGGG**AGAACT**TAACAGCGGGTAGTTCG**TAATT**TGCAA
ScoSPL13	TGGGG**ATGAAT**TAGTTCCGACCCGAATT**CTGTT**ATTCCG
ScoSPL20	TGAGC**TGGTAG**ACGAAGGCGCCCGAGTC**TCCTT**CGTTC
ScoSPL25	GCTGG**TAGACG**AAGGCGCCCGAGTCTCC**TTCGTT**CACTG
ScoSPL50	GAGGT**TTGCGA**ATGTCGCCTTGGTGCCC**TTGTT**CGTCA
ScoSPL51	CATAT**TTTGAT**TATTCTTTTTCTTTTTC**TTCTT**CTCTG
ScoSPL52	GTGCC**TTGTGC**TTTGATTGGCTTTATCG**TTGTT**GTGGC
ScoSPL110	GGCAA**GTGTTC**AGAGATAAAGGGCTCGG**TTATT**AGGGA
ScoSPL117	TGCTC**GCTGTT**TTTTTACGTCTTGGGGA**TTACT**GGAAC
ScoSPL185	TGGCC**CCGACC**AGGCTCGCTCCCCCATT**TTTAC**CTCGG

## Discussion

Gene overexpression and gene deletion have been the most common strategies used in metabolic engineering to improve flux through a desired pathway. However, these strategies have some drawbacks. Deletion of a gene leading to an undesired product may lead to diminished performance, due to its involvement in some important function in the cell [37]. Overexpression of genes often leads to excessive burden on the cell [38]. Hence the SPL concept is attractive in order to tune expression levels for optimal outcome.

Different approaches have been proposed to improve ACT production in *S. coelicolor*. Wang *et al.* (2008) report increased ACT production in *S. coelicolor* by introducing a mutation conferring drug resistance to streptomycin, rifampin and so on. They report production of 1.63 g/i ACT in mutants that harbored 7 or 8 mutations that conferred resistance to 7 or 8 antibiotics. [39]. In another approach, Craney *et al*. (2012) screened a library of small chemical compounds that enhance ACT production. ACT production in this approach increased by up to 5-fold [40] but final concentration achieved is not reported. In our approach, we modulated ACT production by randomizing the promoter sequence of a positive regulator *actII orf4* and report ACT concentration of 3 g/l and i.e. up to 3-fold increase in ACT production compared to the WT strain. These optimization approaches are not mutually exclusive, and could be combined to yield a more efficient production strain.

In this study, a SPL defined from 200 colonies displaying different expression levels was screened for production of ACT and yield of ACT on biomass. The variability of expression levels generated by the library does not necessarily correlate only to different promoter strengths. This approach relies on the “survival window” to screen against small colonies in which the promoter negatively affects cell viability. For example, SPLs may contain some very strong promoters, but if strong overexpression of the target gene is toxic, these promoters will not give viable cells, and will be eliminated from the screen. Even for non-toxic proteins, if the promoter is too strong, and the protein folding capacity of the cell is not sufficient to match it, this will also result in diminished protein levels, and loss of correlation between promoter strength and detectable expression. All SPL colonies that grew on the minimal medium in microtiter plates contained promoters yielding viable expression levels of *actII orf4*. When the 200 selected colonies were inoculated in the minimal medium, some of the colonies failed to grow. This could be due to high expression levels of *actII orf4*, which had negative effect on the cell growth in the minimal medium, where it needs to synthesize all building blocks for biomass formation *de novo*.

We selected 11 SPL strains for detailed physiological characterization, promoter sequencing and determination of the expression level using a reporter gene. These strains showed varied ACT production, and correspondingly, exhibited different expression levels i.e. lower and higher than the expression level of the native *actII orf4* promoter. SPLs constructed for *S. cerevisiae* [19], *E. coli* [21], *L. lactis* [14] and *S. lividans* [24] also reported broad spectra of expression from the synthetic promoters (several orders of magnitude). In those cases, the promoters could be used for optimizing gene expression of other genes in the same organism or in the same genus (P.R. Jensen, personal communication).

Among the 11 selected strains, most of those that showed higher yield of ACT on biomass also demonstrated higher expression levels of the reporter gene (ScoSPL9, ScoSPL25, ScoSPL51, ScoSPL52 and ScoSPL110). In these strains, despite the higher amount of produced ACT, lower final concentration of biomass was observed, indicating that they underwent stress during growth (detectable in their growth behavior). The ScoSPL20 promoter was an exception to the ranking order. It showed similar expression level of the reporter gene to the WT, but its ACT yield on biomass was considerably higher. Surprisingly, in the ScoSPL20 strain ACT production started already in the exponential growth phase. The ScoSPL20 strain did not seem to exhibit any stress during growth. We speculate that the stress recorded in all the SPL strains except for ScoSPL20 may be related directly (cost of protein synthesis) or indirectly (consequence of deregulation of ActII orf4 target genes) to the burden of higher expression of *actII orf4*. Strains with lower yield of ACT on biomass compared to the WT demonstrated lower expression levels of the reporter gene and also produced little or no ACT.

In the construction of a generalized promoter library for *Streptomyces* spp. Seghezzi *et al*. (2011) reported a relatively conserved −35 region, but a considerable variation in the −10 region. Stronger promoters corresponded to enrichment in G within the promoter sequence [24]. Interestingly, in our case, the promoter sequencing revealed extensive loss of conservation in both the −10 and −35 region defined in the primers used for SPL construction. The ScoSPL20 contained two extra G residues in the −35 region compared to the wild type *actII orf4* promoter. This may reflect the fact that the expression of *actII orf4* is incompatible with the consensus sequences we used, and the library screening selected mutated promoters that are viable in combination with the gene *actII orf4*.

Comparison of gene expression profiles of the WT and the ScoSPL20 strains demonstrated interesting effects. Intra-strain comparisons, i.e. exponentially growing WT to ACT-producing WT and exponentially growing ScoSPL20 to ACT-producing ScoSPL20 resulted in 299 and 449 differently expressed genes, respectively. We classified genes in various groups and it was evident from the comparison of exponentially growing ScoSPL20 to ACT-producing ScoSPL20 that protein synthesis and central carbon metabolism (glycolysis, PP pathway and TCA cycle) were substantially reduced in stationary phase as expected. However, the number of genes down-regulated in both gene classes was significantly higher in the ScoSPL20 than in the WT. This might be correlated to the increased glucose uptake rate (−r_S_) in the ScoSPL20. Increased glycolytic flux and TCA cycle activity in the exponential phase due to the increased uptake rate [41] would result in a more significant difference between exponential and ACT production phase with regard to down-regulation of these genes when primary metabolism ceases.

We observed that the RED production was not severely affected in the ScoSPL20 strain. This is in agreement with the findings in the *S. coelicolor* oxp-*actII orf4* strain, where we also observed simultaneous onset of RED and ACT production. This notion is also supported by the transcriptome analysis. However, our results are inconsistent with earlier observations of Huang *et al*. (2005). They reported a relative decrease in *red*-independent genes of the RED cluster as a response to *actII orf4* overexpression [42]. Their studies were based on the R5 agar medium, while we used phosphate limited liquid minimal medium and the disagreement may be a result of this difference. We found 25 genes involved in ACT biosynthesis that were up-regulated in the stationary phase for both the WT and the ScoSPL20 strain, while 18 and 24 genes involved in RED biosynthesis were up-regulated in ACT-producing phase in the WT strain and the ScoSPL20, respectively. This indicates that overexpression of *actII orf4* does not have negative regulatory effects on RED synthesis.

Comparison of the gene expression profiles of ACT-producing WT to ACT-producing ScoSPL20 pin-pointed 154 genes that were differentially expressed in the ScoSPL20. Of these, 15 genes involved in ACT biosynthesis were up-regulated in the ScoSPL20. This is coherent with the increased ACT production observed in the ScoSPL20 compared to the WT strain. In addition, genes encoding NADH dehydrogenase subunits and genes for glutamate metabolism were up-regulated in the ScoSPL20 strain. Biosynthesis of ACT requires 12 ATP and 6 NADPH molecules [43]. Hence up-regulation in NADH dehydrogenase and glutamate metabolism may be connected to requirements for precursors, i.e. ATP (NADH dehydrogenase) and NADPH (glutamate metabolism) for ACT biosynthesis.

In addition to genes directly involved in antibiotic biosynthesis, we observed up-regulation of other genes (hypothetical or putative) that are related to nitrogen and phosphate metabolism when comparing the expression patterns for exponential and stationary phase for both strains. Several of these genes have also previously been shown to be connected to ACT biosynthesis [44], [45], [46]. They include the alkaline phosphatase SCO2286, and the glycerophosphoryl diester phosphodiesterase (*glpQ*) homologs SCO1968 and SCO7750, as previously observed by Lian *et al.*, 2008 [44]. For nitrogen metabolism, *glnA* (SCO2198) that plays a key role in nitrogen assimilation at low ammonia concentration was up-regulated in the stationary phase in both strains. Several other genes with significantly different expression belonged to nitrogen and phosphate metabolism categories, confirming earlier reports on interplay between nitrogen and phosphate metabolism and regulation of ACT biosynthesis [44].

The *S. coelicolor* oxp-*actII orf4* strain that served as a reference strain in this study contained *actII-orf4* expressed under a strong constitutive promoter. This strain had a decreased biomass yield and only a slightly elevated ACT productivity compared to the WT strain, indicating a high burden on the cell. In contrast, the ScoSPL20 promoter that showed similar activity to the WT promoter but was expressed earlier in the growth phase, showed comparable biomass yields as the WT strain while gaining an almost three-fold higher ACT productivity. This highlights the utility of fine-tuning of gene expression as an interesting alternative to overexpression for improving product formation [19].

## Supporting Information

Figure S1 **Plasmid construction.** Different steps carried out in construction of plasmid pGM160_up_*actII orf4* and pGM160_SPL that are used in construction synthetic promoter library.
(TIF)

Figure S2 **Selection of ScoSPL colonies.** Flow chart describing selection of ScoSPL colonies from conjugation plates.
(TIF)

File S1 Includes detailed protocols to support methods and materials section. Different primer sequences designed for this study are also included here.
(DOC)
