# Supplementary figures and images for: Synthetic Promoter Library for Modulation of Actinorhodin Production in Streptomyces coelicolor A3(2)
Source: PLoS One. 2014 Jun 25;9(6):e99701. doi: 10.1371/journal.pone.0099701 (PMC4070896; doi:10.1371/journal.pone.0099701)

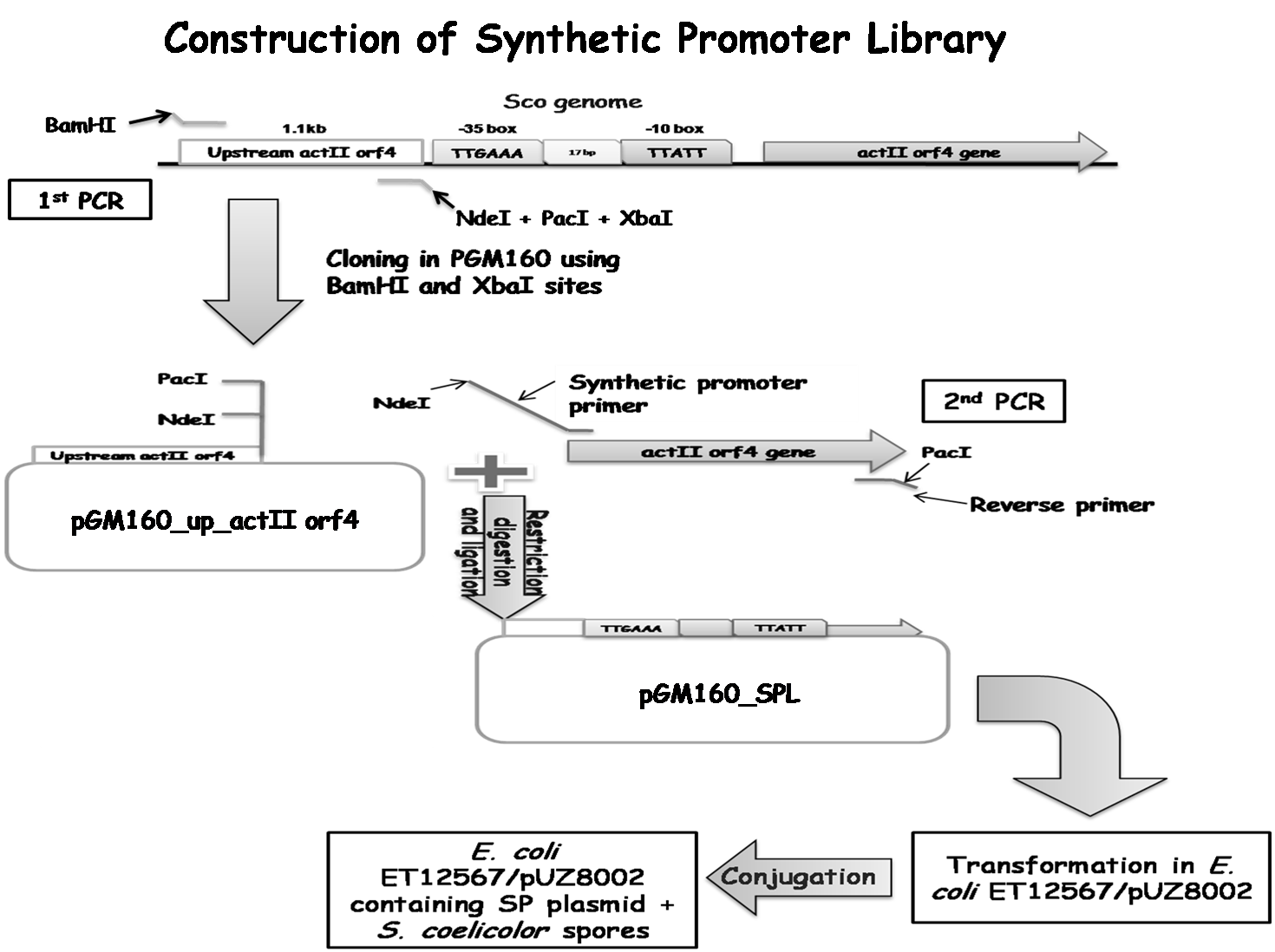

Supplement: Figure S1 — Plasmid construction. Different steps carried out in construction of plasmid pGM160_up_actII orf4 and pGM160_SPL that are used in construction synthetic promoter library. (TIF) [file pone.0099701.s001.tif]

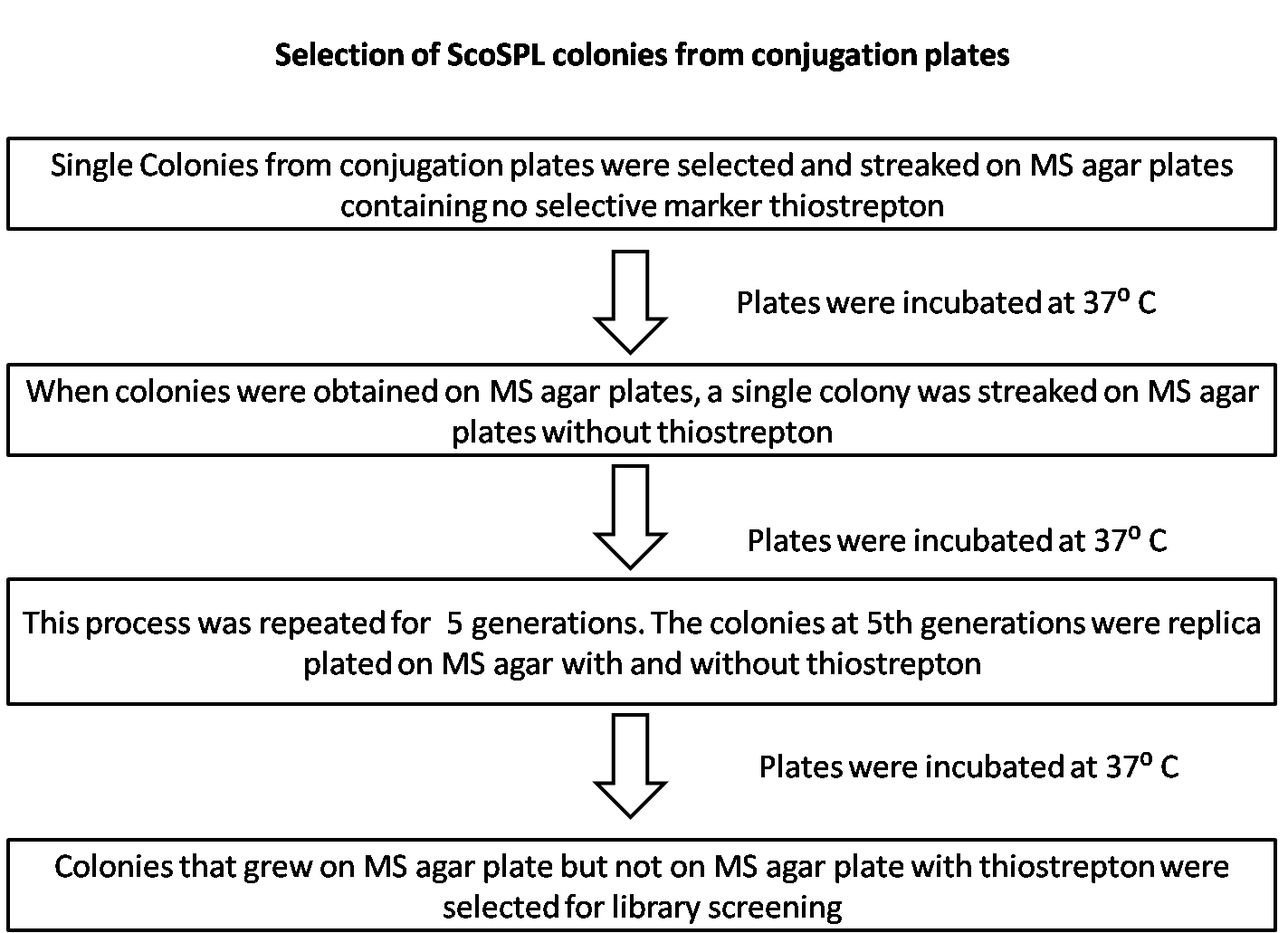

Supplement: Figure S2 — Selection of ScoSPL colonies. Flow chart describing selection of ScoSPL colonies from conjugation plates. (TIF) [file pone.0099701.s002.tif]
