# Supplementary material for: Synthetic Promoter Library for Modulation of Actinorhodin Production in Streptomyces coelicolor A3(2)
Source: PLoS One. 2014 Jun 25;9(6):e99701. doi: 10.1371/journal.pone.0099701 (PMC4070896; doi:10.1371/journal.pone.0099701)
Supplement: File S1 — Includes detailed protocols to support methods and materials section. Different primer sequences designed for this study are also included here. (DOC) [file pone.0099701.s003.doc]

***Supporting Information***

**Table S1: Bacterial Strains and plasmids** used in the study

| **Strain/Plasmid** | **Source** | **Use** |
| --- | --- | --- |
| *Escherichia coli* DH5α |  | Used for routine transformations |
| *Escherichia coli* strain ET12567/pUZ8002 | *(Macneil et al., 1992)* | used for transferring the plasmid DNA into *S. coelicolor* A3(2) |
| *Streptomyces coelicolor* A3(2) strain (SCP1^-^ and SCP2^-^) | *gift from Professor Mervyn Bibb (John Innes Center, UK)* | *Used as a model organism in this study* |
| *Plasmid pIJ10257* | *John Innes Centre, UK* | *Used for construction of actII orf4* overexpression strain of *S. coelicolor* |
| *Plasmid pGM160* | *Prof. Gunther Muth, University of Tubingen, Germany*  (Mu*th et a*l., 1989) | Used in construction ofreplacement of wild type *actII orf4* promoter with a synthetic promoter on the chromosome |
| Plasmid pIJ486-xeg, harboring the xyloglucanase gene | *Dr. KristofVrancken, University of Leuven, Belgium* | xeg used as a reporter gene to determine promoter strength |

***Methods details used in the study***

***Table S2: Primer sequences used in construction of S. coelicolor oxp-actII orf4***

| Forward primer with Nde I site | 5’- TGCGACATATGATGAGATTCAACTTATTGGGACGTGTC -3’ |
| --- | --- |
| Reverse primer with PacI site | 5’- GTCATTAATTAACTACACGAGCACCTTCTCACCGTTGAG -3’ |

**Text S1: Frozen mycelium stok preparation for SPL colonies**

Each *Streptomyces* colony was inoculated in 3 ml 2X YT medium in a 24 well microtiter plate (MTP) with working volume of 3 ml and streaked on MS agar plate to make spore stocks. MTPs were incubated at 28° C for 4 days at 150 rpm. Shaker speed was increased to 250 rpm 30 minutes before harvesting. Each sample was then divided in two aliquots of 1.5 ml. Both aliquots were centrifuged at 16000 X g for 5 minutes and the supernatant was discarded. One aliquot was used for OD_450nm_ measurement after mixing it with 1 ml of 0.9% saline. 1 ml of 20% peptone was added to the remaining aliquot, which was preserved at -20° C.

**Text S2: Preparation of minimal medium used in characterization of SPL**

The minimal medium contained 100 mM NH_4_Cl, 3 mM NaH_2_PO_4_ and 30 g L^-1^ glucose. 100 mM 3-(N-morpholino) propanesulfonic acid (MOPS) was used as a buffer. All the components, except glucose and vitamins, were mixed in 880 ml distilled water and autoclaved. Glucose and vitamins were added after autoclaving. pH of the medium was adjusted to 6.85 after autoclavation using sterile 4 M NaOH. The volume was made up to 1000 ml.

**Text S3: Transcriptome Analysis**

a) Sampling and RNA isolation

Samples for RNA isolation were collected from bioreactors. 20 ml of sample was rapidly mixed with 25 ml of crushed ice and cells were then pelleted at 4000 rpm for 5 minutes, supernatant was discarded. The pellet was then instantly frozen in liquid nitrogen and stored at -80° C.

Total RNA extraction was performed using Qiagen RNeasy mini kit (Hilden, Germany) according to manufacturer’s instructions. Quality and integrity of RNA samples prior to hybridization was assessed with Agilent 2100 bioanalyzer (Santa Clara, USA) and RNA 6000 Nano Labchip kit (Santa Clara, USA).

b) Probe preparation and hybridization of arrays

10 µg of total RNA was used as starting material for cDNA synthesis. cDNA synthesis, fragmentation and labeling of cDNA and hybridization to the affymetrix chip was performed as per the instructions in the Affymetrix manual (Affymetrix,2000). Genechip fluidics station 450 (Santa Clara, USA) was used for staining and washing the arrays. Arrays were scanned using AffymetrixGeneArray scanner 3000 7G (Santa Clara, USA).

**Table S3:** Sequences of primers used to clone Synthetic Promoter upstream of Xeg gene

| **Name of the primer** | **Sequence** |
| --- | --- |
| **SPL7_xeg_fw** | TCGATCTAGACGGGCTGGGGCTCGGGGCCGCCGGTGAGCTGGTAGACGAAGCTGTACAGGGGACAGCTGGGACACCCAAGGAAGAAGGCTGACGTCCGACATGCGTCGCACCCTCAAG |
| **SPL9_xeg_fw** | TCGATCTAGAGCGGGAGAACTTAACAGCGGGTAGTTCGTAATTTGCAAAGCTGTACAGGGGACAGCTGGGACACCCAAGGAAGAAGGCTGACGTCCGACATGCGTCGCACCCTCAAG |
| **SPL13_xeg_fw** | TCGATCTAGAGGGGATGAATTAGTTCCGACCCGAATTCTGTTATTCCGCCAGCTGTACAGGGGACAGCTGGGACACCCAAGGAAGAAGGCTGACGTCCGACATGCGTCGCACCCTCAAG |
| **SPL20_xeg_fw** | TCGATCTAGATGAGCTGGTAGACGAAGGCGCCCGAGTCTCCTTCGTTCAGCTGTACAGGGGACAGCTGGGACACCCAAGGAAGAAGGCTGACGTCCGACATGCGTCGCACCCTCAAG |
| **SPL25_xeg_fw** | TCGATCTAGAGCTGGTAGACGAAGGCGCCCGAGTCTCCTTCGTTCACTGAGCTGTACAGGGGACAGCTGGGACACCCAAGGAAGAAGGCTGACGTCCGACATGCGTCGCACCCTCAAG |
| **SPL50_xeg_fw** | TCGATCTAGAGAGGTTTGCGATGTCGCCTTGGTGCCCTTGTTCGTCAAGCTGTACAGGGGACAGCTGGGACACCCAAGGAAGAAGGCTGACGTCCGACATGCGTCGCACCCTCAAG |
| **SPL51_xeg_fw** | TCGATCTAGACATATTTTGATTATTCTTTTTCTTTTTCTTCTTCTCTGAGCTGTACAGGGGACAGCTGGGACACCCAAGGAAGAAGGCTGACGTCCGACATGCGTCGCACCCTCAAG |
| **SPL52_xeg_fw** | TCGATCTAGAGTGCCTTGTGCTTTGATTGGCTTTATCGTTGTTGTGGCAGCTGTACAGGGGACAGCTGGGACACCCAAGGAAGAAGGCTGACGTCCGACATGCGTCGCACCCTCAAG |
| **SPL110_xeg_fw** | TCGATCTAGAGGCAAGTGTTCAGAGATAAAGGGCTCGGTTATTAGGGAAGCTGTACAGGGGACAGCTGGGACACCCAAGGAAGAAGGCTGACGTCCGACATGCGTCGCACCCTCAAG |
| **SPL117_xeg_fw** | TCGATCTAGATGCTCGCTGTTTTTTTACGTCTTGGGGATTACTGGAACAGCTGTACAGGGGACAGCTGGGACACCCAAGGAAGAAGGCTGACGTCCGACATGCGTCGCACCCTCAAG |
| **SPL179_xeg_fw** | TCGATCTAGATGAGCTGGTAGACGAAGGCGCCCGAGTCTCCTTCGTTCAGCTGTACAGGGGACAGCTGGGACACCCAAGGAAGAAGGCTGACGTCCGACATGCGTCGCACCCTCAAG |
| **SPL185_xeg_fw** | TCGATCTAGATGGCCCCGACCAGGCTCGCTCCCCCATTTTTACCTCGGAGCTGTACAGGGGACAGCTGGGACACCCAAGGAAGAAGGCTGACGTCCGACATGCGTCGCACCCTCAAG |
| ***actII orf4* wt_xeg_fw** | TCGATCTAGAGCACATTGAAATCTGTTGAGTAGGCCTGTTATTGTCGCCCCCAGGAGACGGAGAATCTCGACGGGGGCGCAGATGCGTCGCACCCTCAAG |
| **Xeg_rv** | ATCCATGGTCATATGACTAACCTCTAGTC |

*** Grey shaded part represents Xeg gene homology sequence**
